# Downregulation of the NLRP3 inflammasome by adiponectin rescues Duchenne muscular dystrophy

**DOI:** 10.1186/s12915-018-0501-z

**Published:** 2018-03-20

**Authors:** Raphaël Boursereau, Michel Abou-Samra, Sophie Lecompte, Laurence Noel, Sonia M. Brichard

**Affiliations:** 1 Endocrinology, Diabetes and Nutrition Unit, Institute of Experimental and Clinical Research, Medical Sector, Catholic University of Louvain, 1200 Brussels, Belgium; 2 IREC – Endocrinology, Diabetes and Nutrition Unit, UCL/EDIN B1.55.06 – Av. Hippocrate 55, Harvey 55, B-1200 Brussels, Belgium

**Keywords:** Adiponectin, miR-711, NLRP3 inflammasome, Skeletal muscle, Inflammation, Duchenne muscular dystrophy

## Abstract

**Background:**

The hormone adiponectin (ApN) exerts powerful anti-inflammatory effects on skeletal muscle and can reverse devastating myopathies, like Duchenne muscular dystrophy (DMD), where inflammation exacerbates disease progression. The NLRP3 inflammasome plays a key role in the inflammation process, and its aberrant activation leads to several inflammatory or immune diseases. Here we investigated the expression of the NLRP inflammasome in skeletal muscle and its contribution to DMD.

**Results:**

We find that NLRP3 is expressed in skeletal muscle and show that ApN downregulates NLRP3 via its anti-inflammatory mediator, miR-711. This repression occurs both in vitro in C2C12 myotubes and in vivo after either local (via muscle electrotransfer) or systemic (by using transgenic mice) ApN supplementation. To explore the role of the NLRP3 inflammasome in a murine model of DMD, we crossed mdx mice with Nlrp3-knockout mice. In mdx mice, all components of the inflammasome were upregulated in muscle, and the complex was overactivated. By contrast, in mdx mice lacking Nlrp3, there was a reduction in caspase-1 activation, inflammation and oxidative stress in dystrophic muscle, and these mice showed higher global muscle force/endurance than regular mdx mice as well as decreased muscle damage. To investigate the relevance of NLPR3 regulation in a human disease context, we characterized NLRP3 expression in primary cultures of myotubes from DMD subjects and found a threefold increase compared to control subjects. This overexpression was attenuated by ApN or miR-711 mimic treatments.

**Conclusions:**

The NLRP3 inflammasome plays a key pathogenic role in DMD and muscle inflammation, thereby opening new therapeutic perspectives for these and other related disorders.

**Electronic supplementary material:**

The online version of this article (10.1186/s12915-018-0501-z) contains supplementary material, which is available to authorized users.

## Background

Chronic muscle inflammation may be present as either a low-grade or a severe form. The low-grade form characterizes obesity-linked metabolic disorders [1, 2]. A more aggressive form may play a role in the development of myopathy and in disease severity. Duchenne muscular dystrophy (DMD) is the most frequently inherited human myopathy and the most devastating type of muscular dystrophy. DMD stems from X-linked recessive defects in the gene encoding for dystrophin, a protein that provides structural stability and integrity to the myofibre membrane**.** Absence of this protein leads to membrane damage, allowing massive infiltration of immune cells, chronic inflammation, necrosis and severe muscle degeneration [3]. Although dystrophin mutations represent the primary cause of DMD, it is the secondary processes involving chronic and severe inflammation that likely exacerbate disease progression [4, 5].

Adiponectin (ApN) is a hormone abundantly secreted by adipocytes under normal conditions. Circulating ApN is significantly decreased in obese individuals and in patients with criteria for the metabolic syndrome [6]. Besides its metabolic (mainly insulin-sensitizing and fat-burning) properties, ApN has emerged as a master regulator of inflammation/immunity in a variety of tissues including the skeletal muscle [7, 8]. ApN turned out to be sufficiently powerful to offset severe inflammation/oxidative stress and muscle damage in dystrophic muscles of mdx mice (a model of DMD). This was demonstrated in our very own mouse model (mdx-ApN), where mdx animals were crossed with transgenic mice overexpressing ApN [9]. Conversely, ApN deficiency worsened the mdx phenotype, while muscle electrotransfer of the ApN gene reversed inflammation/oxidative stress and disease progression, thereby suggesting a therapeutic potential for ApN in DMD [10].

**Fig. 1:**
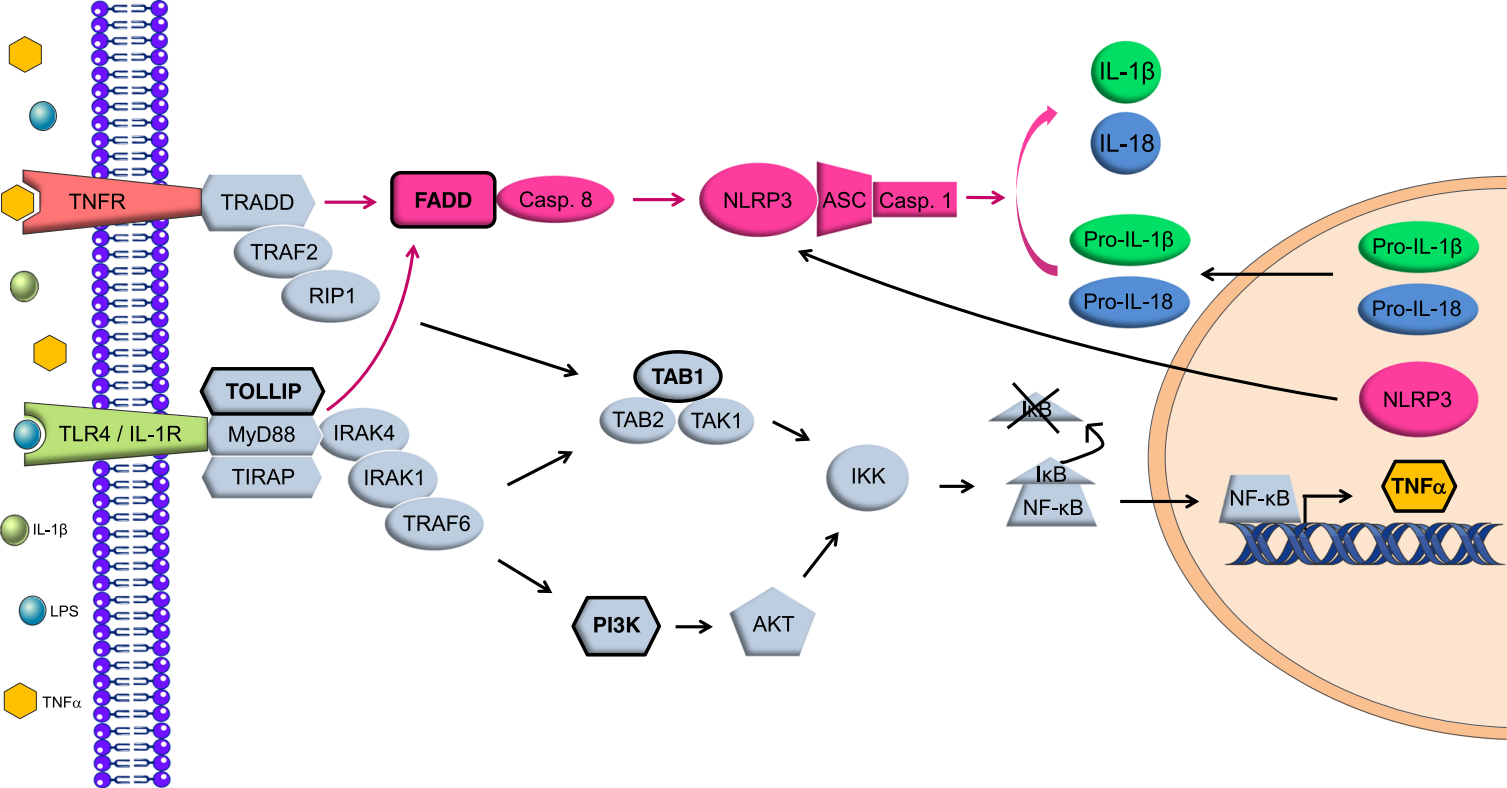
Inflammation/immune signalling pathways repressed by miR-711 in muscle. The target genes of miR-711 are indicated in *bold* and outlined in *black*. They are involved in inflammatory/immune signalling pathways that split into two branches. The first one, depicted in *grey*, has been recently described [11] and results in NF-κB activation. Briefly, binding of LPS or IL-1β to their respective receptor TLR4 or IL-1R triggers the assembly of a complex (composed of MyD88 and TOLLIP), while activation of TNFR signalling by TNFα triggers the assembly of another complex including TRADD. These intracellular scaffolds stimulate the IKK complex through activation of TAB1 or PI3K. Next, the IKK complex releases NF-κB from its repressor IκB, thereby permitting its translocation into the nucleus. This in turn upregulates transcription of pro-inflammatory factors such as TNFα and inflammasome targets/components like pro-IL-1β/-18 and NLRP3 (i.e. priming of NLRP3). The second branch in *fuchsia* leads through FADD and caspase-8 activation to NLRP3 activation. FADD can be recruited by the TRADD or MyD88 complex. Inflammasome activation involves assembly of NLRP3 with ASC and pro-caspase-1. This complex triggers pro-caspase-1 self-cleavage into active caspase-1, which then cleaves pro-IL-1β and pro-IL-18 into their biologically active cytokines. Abbreviations: *AKT* protein kinase B, *ASC* apoptosis-associated speck-like protein, *FADD* Fas-associated protein with death domain, *IκB* inhibitor of kappa B, *IKK* IκB kinase, *IL-1R* interleukin-1 receptor, *IRAK1/4* interleukin-1 receptor-associated kinase 1/4, *LPS* lipopolysaccharide, *MyD88* myeloid differentiation primary response 88, *NF-κB* nuclear factor kappa B, *PI3K* phosphatidylinositol-4,5-bisphosphate 3-kinase, *RIP1* receptor interacting protein 1, *TAB1* transforming growth factor beta (TGF-β) activated kinase 1-binding protein 1, *TAK1* TGF-β activated kinase 1, *TIRAP* Toll-interleukin 1 receptor domain-containing adapter protein, *TLR4* Toll-like receptor 4, *TNFR* tumour necrosis factor receptor, *TOLLIP* Toll interacting protein, *TRADD* TNFR type 1-associated death domain protein, *TRAF2/6* TNFR associated factor 2/6

We have also recently shown that the anti-inflammatory action of ApN on skeletal muscle was at least in part mediated by a micro RNA (miRNA), miR-711. Thus, ApN upregulated miR-711 in muscle, which in turn repressed genes belonging to inflammation/immunity signalling cascades [11] (these genes are indicated in bold in Fig. 1). These cascades split into two branches. The first branch, which has been described recently, leads to nuclear factor kappa B (NF-κB) activation [11]. The second branch, still undescribed, could potentially lead to NLRP3 inflammasome stimulation. Fas-associated protein with death domain (FADD) is indeed one of the target genes of miR-711, and this protein serves as an apical mediator of both priming and activation of NLRP3 inflammasome through the activation of caspase-8 [12, 13].

The inflammasome is involved in the initiation or progression of diseases with a high impact on public health, such as metabolic disorders and neurodegenerative diseases [14]. The best characterized inflammasome is NLRP3, so named because the NLRP3 protein belongs to the family of nucleotide-binding and oligomerization domain-like receptors (NLRs). NLRP3 must be primed before activation. NLRP3 priming results from activation of NF-κB, which in turn upregulates transcription of inflammasome components/targets, including inactive NLRP3, pro-interleukin (IL)-1β and pro-IL-18 [14, 15] (see Fig. 1). Next, activation involves NLRP3 binding to apoptosis-associated speck-like protein (ASC) and pro-caspase-1, forming a complex termed the inflammasome. This triggers pro-caspase-1 self-cleavage into active caspase-1, which then cleaves pro-IL-1β and pro-IL-18 to their active forms [14, 15]. So far, the NLRP3 inflammasome has been poorly studied in muscle [16, 17].

The aims of this work were first to study whether NLRP3 was present in skeletal muscle, more specifically within myofibres, and second to test whether it was regulated by ApN through the miR-711. Third, we investigated whether NLRP3 may play a crucial role in the pathogenesis of DMD. For this matter, we crossed mdx mice with Nlrp3-knockout (Nlrp3-KO) mice to generate mdx mice with NLRP3 depletion (mdx/Nlrp3-KO). Finally, we translated our data to humans.

## Results

### NLRP3 inflammasome is present within myofibres

**Fig. 2:**
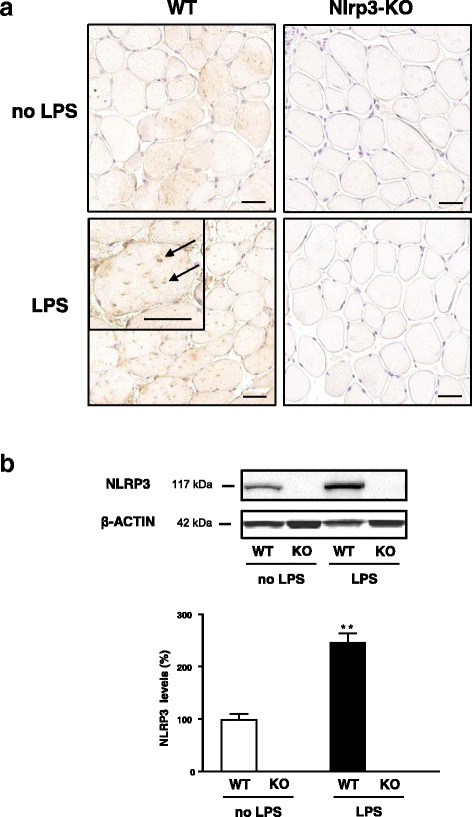
Evidence for NLRP3 expression within mouse myofibres. Wild-type mice (WT) and Nlrp3-KO mice were challenged by intraperitoneal (ip) LPS or received vehicle only (no LPS), and *tibialis anterior* muscles were sampled 24 h later. **a** Immunochemistry was performed with a specific antibody directed against NLRP3. Clusters of staining, shown by *arrows* in the magnification *inset*, were detected in myofibres of WT mice after LPS, while there was no labelling in Nlrp3-KO mice. Mice receiving vehicle only are shown for comparison. Scale bar = 50 μm for all images including the inset. Representative sections for six mice per group are presented. **b** Western blot analysis was also performed in the different groups of mice; data were normalized to actin levels and presented as percentages of WT without LPS. Values are means ± standard error of the mean (SEM) for three to six mice per group. **P < 0.01 for the effect of LPS

We first provided evidence for NLRP3 expression in myofibres. To this end, wild-type (WT) and Nlrp3-KO mice were challenged by intraperitoneal (ip) injection of lipopolysaccharide (LPS) to induce inflammation or received vehicle only. In basal conditions (no LPS), NLRP3 staining was faint in WT mice and undetectable in Nlrp3-KO ones (Fig. 2). After LPS, NLRP3 was detected as stained clusters in the sarcoplasm of WT mice, while there was still no labelling in Nlrp3-KO muscle (Fig. 2). These results were confirmed by Western blot analysis. Taken together, our data showed that NLRP3 is produced by mouse myofibres and could play an important role in muscle inflammation.

### NLRP3 expression is regulated by ApN and miR-711 as well as by FADD in murine myotubes

**Fig. 3:**
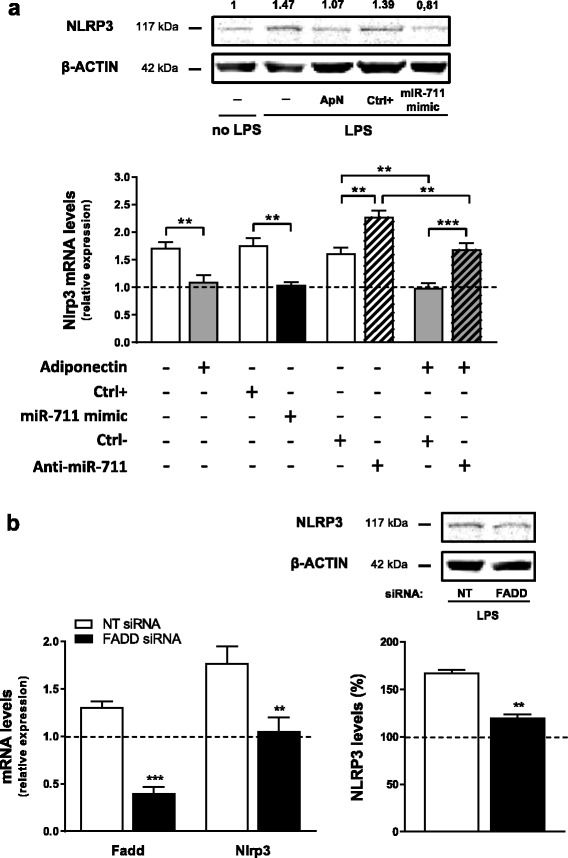
Effects of ApN, miR-711 (**a**) and FADD (**b**) on NLRP3 expression in vitro. **a** C2C12 myotubes were either treated or not with ApN or transfected with miR-711 mimic or its control (Ctrl+) for 24 h. Cells were also transfected with anti-miR-711 or its relative control (Ctrl–) for 28 h, while ApN was added during the last 24 h. All conditions presented herein were obtained in C2C12 challenged by LPS except for the basal condition (no LPS, no transfection or any other treatments) represented by the *dotted line*. mRNA levels were normalized to cyclophilin, and the subsequent ratios were presented as relative expression compared to basal condition. Values are means ± SEM for seven repeated experiments. A Western blot with five experimental conditions (basal, LPS alone, ApN + LPS, control miR + LPS and miR mimic + LPS) is also shown above the histograms. NLRP3 protein expression was normalized to actin levels and the subsequent ratios presented as relative expression compared to basal condition. This blot is representative of two different experiments; the mean NLRP3 value is indicated at the top of each condition. **b** Effects of FADD silencing on NLRP3 expression in C2C12 cells challenged by LPS. Myotubes were transfected for 24 h with small interfering RNAs (siRNAs) against FADD or a negative (non-targeting, NT) control. Next, cells were challenged by LPS during the last 20 h. mRNA levels were normalized and presented as relative expression compared to the basal condition (no LPS, no transfection) represented by the *dotted line*. NLRP3 protein expression was measured by Western blot, normalized to actin levels, and also presented as percentages of the basal condition. Values are means ± SEM for five (mRNA) and three (protein) repeated experiments. **P < 0.01, ***P < 0.001 for indicated conditions (**a**) or *vs* NT siRNA (**b**)

We next examined whether NLRP3 is regulated by the anti-inflammatory hormone ApN and its mediator miR-711. This hypothesis was first tested in vitro in inflamed myotubes (Fig. 3a). Nlrp3 messenger RNAs (mRNAs) were increased in C2C12 cells challenged by LPS (compare the first white column with basal conditions represented by the dotted line (i.e. no LPS and any other treatments)). Both ApN treatment and miR-711 transfection reversed this stimulation of Nlrp3 expression (first two pairs of histograms: compare grey/black *vs* white column). Likewise, as shown on Western blot, NLRP3 protein levels, which were expressed relative to basal condition (no LPS), tended to be decreased by both ApN and miR mimic. Conversely, miR-711 silencing further augmented Nlrp3 expression (histograms, 3rd pair of columns). Moreover, the inhibitory effect of ApN on Nlrp3 mRNA was reversed by the anti-miR (last pair of histograms). Taken together, these data suggest that NLRP3 is implicated in muscle inflammation and is downregulated by ApN through miR-711.

To search for mechanisms by which miR-711 may downregulate NLRP3, we studied one of its target genes, FADD, which is known to promote NLRP3 priming and activation [12]. We showed that silencing gene expression of FADD actually repressed Nlrp3 mRNA and protein expression in inflamed myotubes (Fig. 3b), thereby extending data obtained in FADD-deficient macrophages [12].

### Muscle electrotransfer of either ApN complementary DNA (cDNA) or pre-miR-711 prevents LPS upregulation of NLRP3 in mice

We next tested whether this regulation was also effective in vivo. To this end, we took advantage of our previous model, in which local administration of ApN or miR-711 was able to protect muscles of ApN-KO mice against LPS-induced inflammation [11]. Briefly, one *tibialis anterior* muscle was injected with a plasmid containing the ApN sequence (p-ApN) or the pre-miR-711 (p-miR-711), while the contralateral one received an empty plasmid; the muscles were then electroporated. Nine days later, mice were challenged by ip injection of LPS. Muscles were sampled 24 h later.

**Fig. 4:**
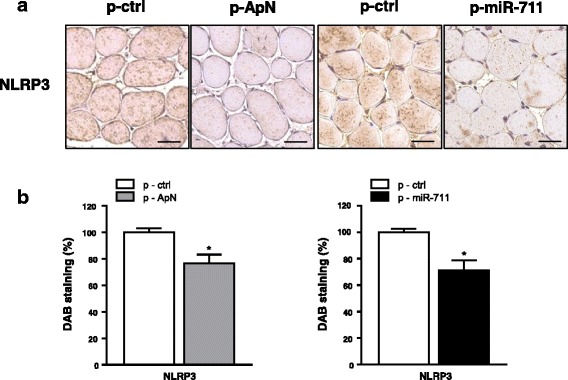
Effects of ApN cDNA and pre-miR-711 electrotransfer on NLRP3 in skeletal muscles of ApN-KO mice. One *tibialis anterior* muscle of ApN-KO mice was injected and electroporated with ApN cDNA-containing plasmid (p-ApN) or with pre-miR-711-containing plasmid (p-miR-711), whereas the contralateral muscle was injected and electroporated with the respective control plasmid (p-ctrl). Nine days later, mice were challenged by LPS and the *tibialis anterior* muscles were sampled 24 h later. **a** Immunochemistry was performed with a specific antibody directed against NLRP3. Scale bar = 50 μm. **b** Quantification of 3,3’-diaminobenzadine (DAB) staining areas within muscles. Results are means ± SEM for two groups of five mice (the contralateral muscle being used as control). *P < 0.05 for the effect of ApN or miR-711

Herein, we studied the expression of NLRP3 by immunochemistry. Muscle electrotransfer of the ApN gene or pre-miR-711 reduced NLRP3 staining by ~ 25–30% (Fig. 4a, b). These data indicate that in inflamed muscle NLRP3 is attenuated by ApN or miR-711 in vivo.

### NLRP3 is upregulated in muscle of dystrophic mice but partially corrected by ApN

**Fig. 5:**
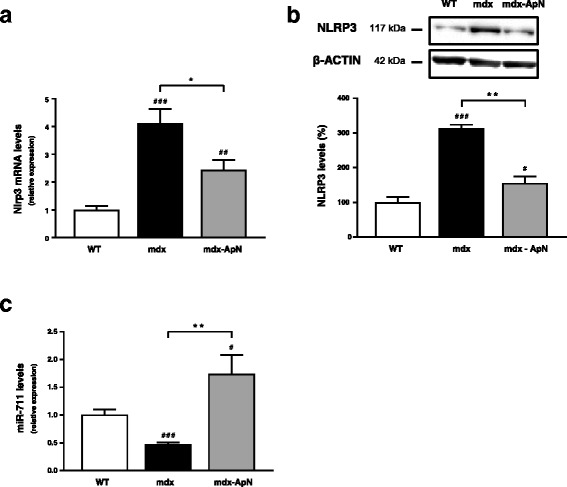
Effects of long-term ApN overexpression on NLRP3 and miR-711 levels in muscles of dystrophic mice. Three groups of mice were used: mdx mice overexpressing ApN, which were generated by transgenesis (mdx-ApN [9]), their mdx littermates (mdx) and WT mice. Nlrp3 mRNA (**a**) and protein (**b**) as well as miR-711 levels (**c**) were measured in *tibialis anterior* muscles, normalized to cyclophilin (**a**, **c**) or to actin (**b**), and the subsequent ratios were presented as relative expression compared to WT values. Results are means ± SEM for three to seven mice per group. #*P* < 0.05, ##*P* < 0.01, ###*P* < 0.001 *vs* WT; *P < 0.05, **P < 0.01 for mdx-ApN *vs* mdx mice

We next explored whether NLRP3 may play a role in the pathogenesis of DMD, where inflammation markedly exacerbates the disease. Nlrp3 mRNA and protein levels were four- and threefold higher in muscles of mdx mice than in WT ones, respectively. However, this upregulation was attenuated in transgenic mdx mice with chronic overexpression of ApN [9] (Fig. 5a, b). Interestingly, gene expression of miR-711, the mediator of ApN, was strongly decreased in mdx mice, while it was increased in mdx mice overexpressing ApN (Fig. 5c). These results suggest that NLRP3, which is inversely related to miR-711 and ApN, could be involved in the pathogenesis of DMD.

### Effects of NLRP3 depletion on physical performance and muscle injury of mdx mice

To further investigate the pathogenic role of the inflammasome in DMD, we generated mdx mice which were Nlrp3-deficient (mdx/Nlrp3-KO). These mice were compared to their mdx littermates (mdx). Nlrp3-KO mice and WT mice were also used for comparison.

**Fig. 6:**
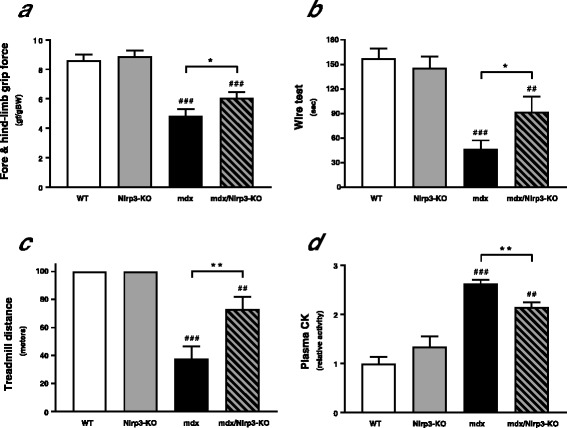
Effects of NLRP3 depletion on global force, resistance and muscle injury in mdx mice. Four groups of mice were used: mdx mice that were also deficient for Nlrp3 (mdx/Nlrp3-KO), regular mdx (mdx), Nlrp3-KO mice and WT ones. Several functional in vivo studies were carried out. **a** The animals were lowered on a grid connected to a sensor to measure the muscle force of both fore- and hindlimbs; data were then expressed in gram-force relative to body weight. **b** Mice were next subjected to a wire test where they were suspended by their forelimbs and the time until they completely released the wire and fell down was recorded. **c** Mice were also submitted to a downhill treadmill exercise for 10 min; the covered distance (metres) was measured for each mouse, 100 m being the maximal distance. **d** Muscle injury was assessed by plasma creatine kinase (*CK*) activity measured in the basal state and expressed as IU/L. Results are means ± SEM for eight to twelve mice per group. ##*P* < 0.01, ###*P* < 0.001 *vs* WT; *P < 0.05, **P < 0.01 for the indicated conditions

These four groups of mice were submitted to three different functional tests in order to evaluate the effects of NLRP3 depletion on muscle force and endurance. The grip test quantitatively measures the global force in limb muscles. The strength of combined fore- and hindlimb muscles was ~ 44% lower in mdx than in WT mice, while mdx/Nlrp3-KO mice showed a significant improvement (Fig. 6a). The wire test evaluates muscle force and resistance to fatigue. In this test, the time during which the mouse is suspended on a horizontal wire is recorded. The mdx mice fell down much faster than WT mice, while the mdx/Nlrp3-KO mice stayed on the wire twice as long (Fig. 6b). Eventually, endurance was evaluated by an eccentric exercise. On the 3rd and last day of the exercise, the distance (metres) covered by each mouse was measured. WT mice covered the maximum distance (100 m), while the running distance of mdx mice fell drastically (~ 38 m); that of the mdx/Nlrp3-KO mice was intermediate (~ 73 m) (Fig. 6c). For each exercise, there were no differences between WT and Nlrp3-KO mice.

We next measured in basal conditions creatine kinase activity, a plasma marker of muscle damage. This marker was ~ 2.6 fold higher in mdx than in WT mice, while it declined by more than 20% in mdx/Nlrp3-KO mice (Fig. 6d).

### Effects of NLRP3 depletion on muscle inflammation and oxidative stress in mdx mice

**Fig. 7:**
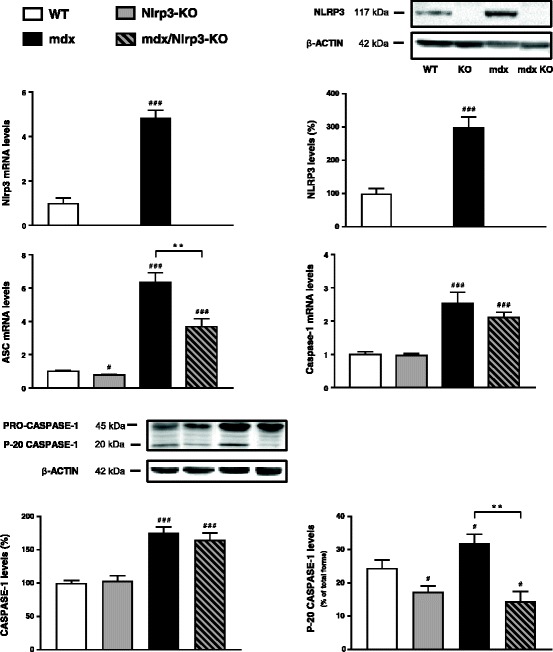
Effects of NLRP3 depletion on components of the inflammasome complex in mdx muscles. *Tibialis anterior* muscles were sampled from WT mice, Nlrp3-KO mice, mdx mice and mdx/Nlrp3-KO mice. Members of the inflammasome complex (NLRP3, ASC and caspase-1) were measured at the mRNA and/or protein levels. mRNA levels were normalized to cyclophilin and presented as relative expression compared to WT values. Protein quantification was performed by Western blot. NLRP3 and total caspase-1 levels (45 kDa proenzyme and its active cleaved subunits) were normalized to actin levels and presented as percentages of WT values. Caspase activity (activated 20 kDa subunit) was expressed as percentage of total forms. Results are means ± SEM for eight to ten (mRNA) or six (protein) mice per group. #*P* < 0.05, ###*P* < 0.001 *vs* WT; **P < 0.01 for indicated conditions

**Fig. 8:**
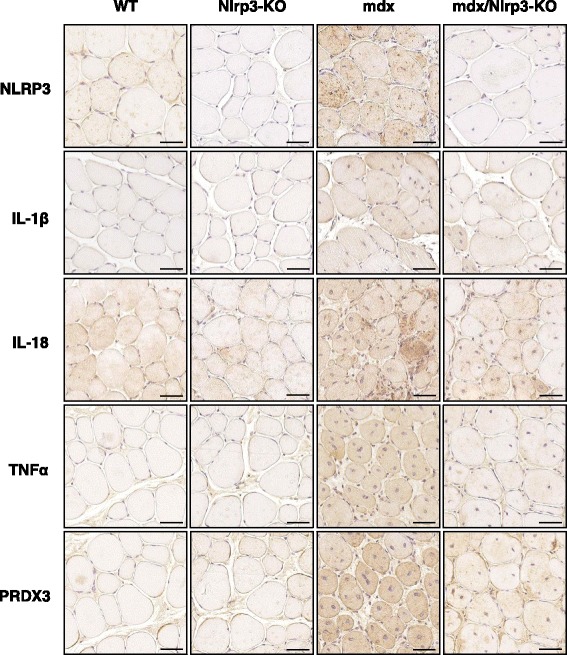
Immunodetection of inflammation and oxidative stress markers in mdx muscles with Nlrp3 abrogation. *Tibialis anterior* muscles were sampled from WT mice, Nlrp3-KO mice, mdx mice and mdx mice that were also Nlrp3-deficient (mdx/Nlrp3-KO). Immunodetection was performed with specific antibodies directed against NLRP3, three pro-inflammatory cytokines (IL-1β, IL-18 and TNFα) and one oxidative stress marker (peroxiredoxin 3, PRDX3). IL-1β antibody was specific for the mature form of the protein, while IL-18 antibody recognized its total form. Representative sections for six mice per group are shown. Scale bar = 50 μm

We first examined the components of the inflammasome complex in the four groups of mice. We confirmed the ablation of the Nlrp3 gene in KO mice (mdx or not) and the upregulation in regular mdx animals (Fig. 7). NLRP3 protein showed a comparable pattern of expression when quantified by Western blotting (Fig. 7) or measurement of 3,3’-diaminobenzadine (DAB) staining areas on immunochemistry sections (like those shown on Fig. 8), the correlation between both techniques being excellent (Additional file 1: Figure S1). Besides Nlrp3, mRNA levels of the adapter protein ASC were also upregulated in mdx mice and partially corrected in mdx/Nlrp3-KO mice (Fig. 7). Gene expression and total protein levels of the last component of this complex, caspase-1, were increased in both regular mdx and Nlrp3-deficient mdx mice. However, caspase-1 activity (percentage cleaved 20 kDa subunits) was upregulated in regular mdx mice only and was actually even downregulated in mdx/Nlrp3-KO mice.

**Fig. 9:**
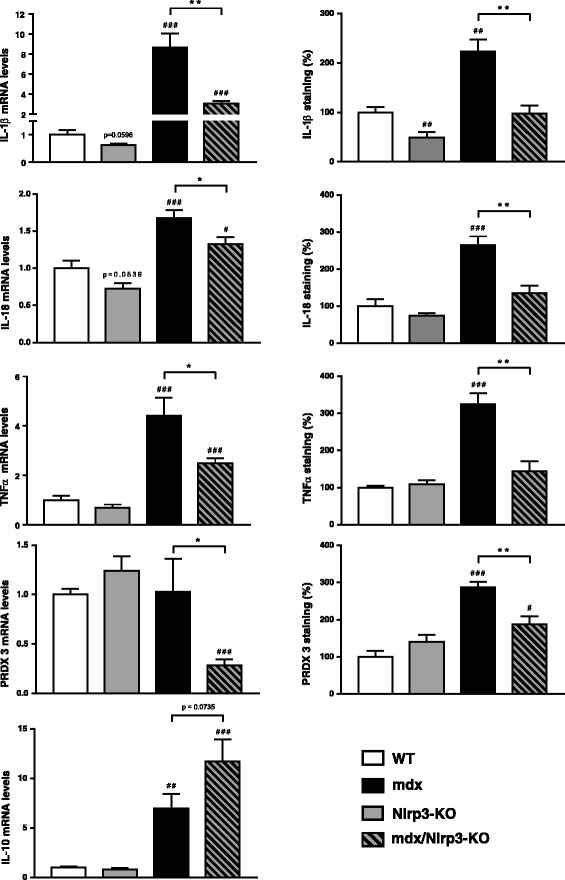
Effects of NLRP3 depletion on different markers of inflammation or oxidative stress in mdx muscles. *Tibialis anterior* muscles were sampled from WT mice, Nlrp3-KO mice, mdx mice and mdx/Nlrp3-KO mice. Three pro-inflammatory cytokines (IL-1β, IL-18 and TNFα), one oxidative stress marker (PRDX3) and one anti-inflammatory cytokine (IL-10) were measured at the mRNA and/or protein levels. mRNA levels were normalized to cyclophilin and presented as the relative expression compared to WT values. Protein quantification was performed by measurement of DAB staining areas on immunochemistry sections (like those shown on Fig. 8), and data were presented as percentages of WT values. Results are means ± SEM for eight to ten (mRNA) or six (protein) mice per group. #*P* < 0.05, ##*P* < 0.01, ###*P* < 0.001 *vs* WT; *P < 0.05, **P < 0.01 for indicated conditions

mRNA and protein levels of caspase-1 targets, IL-1β and IL-18 were upregulated in mdx mice and partially or completely corrected by NLRP3 depletion in dystrophic mice. IL-1β was detected with a specific antibody for its bioactive form and IL-18 with an antibody that recognized its total form (Figs. 8 and 9). Other markers of inflammation (tumour necrosis factor alpha (TNFα)) or oxidative stress (peroxiredoxin 3 (PRDX3)) behaved similarly with a complete or partial correction by Nlrp3 ablation of the otherwise upregulated levels found in mdx mice. Conversely, gene expression of the anti-inflammatory cytokine, IL-10, which was increased in mdx mice likely to compensate inflammation, was further upregulated by Nlrp3 ablation (Fig. 9).

Taken together, these data suggest that abrogation of the inflammasome protects the dystrophic muscle against excessive inflammatory reactions and oxidative stress.

### NLRP3 expression and regulation in human DMD myotubes

**Fig. 10:**
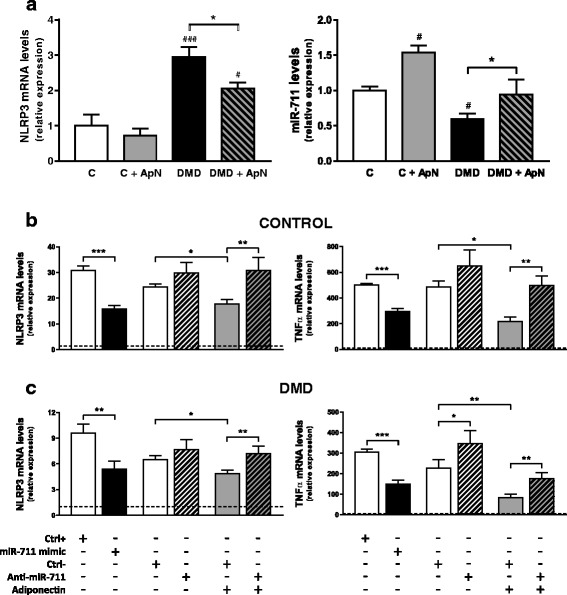
Expression and regulation of NLRP3 in human DMD myotubes. Primary cultures of human myocytes from C (control) and/or DMD subjects. **a** Differentiated myotubes were treated or not with 5 μg/ml ApN for 24 h. Gene expression of human NLRP3 and miR-711 was next quantified, normalized to TATA-box-binding protein (TBP), and the subsequent ratios were presented as relative expression compared to control conditions. Values are means ± SEM for five to six independent cultures (i.e. run at different times and, for each time, from a new vial of cryopreserved myoblasts) from five (NLRP3) or three (miR-711) different subjects in each C and DMD group. The subjects were always chosen at random. #*P* < 0.05, ###*P* < 0.001 *vs* C; *P < 0.05 for indicated conditions. **b**, **c** Differentiated myotubes from C or DMD subjects were challenged by an inflammatory stimulus (a combination of TNFα and interferon gamma (IFNγ)), then transfected or not with miR-711 mimic or anti-miR-711 (or their respective controls: Ctrl+ or Ctrl–), while being treated or not by ApN. mRNA levels of NLRP3 and TNFα were normalized to TBP and presented as the relative expression compared to the basal condition (i.e. no inflammation, no transfection or any other treatments) represented by the *dotted line*. Values are means ± SEM for five to nine independent cultures derived from five different subjects in each C and DMD group. *P < 0.05, **P < 0.01, ***P < 0.001 for indicated conditions

We measured NLRP3 mRNA levels in primary cultured myotubes obtained from healthy controls (C) or from DMD subjects studied in basal conditions (Fig. 10a). NLRP3 mRNA levels were threefold higher in DMD than in control myotubes. This increase was partly attenuated by ApN treatment. miR-711 levels showed a reverse pattern of expression: a 40% decrease in DMD myotubes compared to control ones, while ApN treatment upregulated these levels, thereby leading to their normalization in DMD (Fig. 10a).

We next tested the hypothesis that, as in C2C12 cells, miR-711 may be a mediator of the anti-inflammatory action of ApN in C and DMD myotubes. To mimic the inflammatory microenvironment which prevails in DMD, we challenged the myotubes by an inflammatory stimulus (TNFα/interferon gamma (IFNγ)) [18], as already described [19] (Fig. 10b, c). These cells were then transfected with miR-711 mimic or anti-miR-711 (and their respective controls: Ctrl+ and –) and treated or not with ApN. In C myotubes, NLRP3 expression was markedly upregulated by the inflammatory challenge (first white column *vs* dotted line, which represents basal conditions, i.e. no inflammation and any other treatments; Fig. 10b). Overexpression of miR-711 downregulated NLRP3 mRNAs (compare the first pair of columns; Fig. 10b), while the anti-miR tended to induce an opposite effect (second pair of columns; Fig. 10b). Like the miR-711 mimic, ApN downregulated gene expression of NLRP3 (columns 5 *vs* 3), an inhibition which was reversed by miR-711 silencing (last pair of columns). Changes of TNFα were rather similar to those of NLRP3: downregulation by miR-711 and ApN, upregulation by miR-711 blockade and attenuation of ApN anti-inflammatory effect by this blockade (Fig. 10b). Data obtained in DMD myotubes were qualitatively similar to those in the controls (Fig. 10c). Eventually, as in C2C12 cells, we confirmed that TOLLIP and FADD were also two target genes of miR-711 in both human C and DMD cells (Additional file 2: Figure S2) and could thus be early steps leading to activation of the inflammasome complex.

## Discussion

The inflammasome complex has been well characterized in cells that participate in innate immunity; however, there is still little information about its expression, activation and potential role in non-hematopoietic cells such as skeletal muscle [16, 17]. NLRP3 has been reported to be upregulated in human skeletal muscle biopsies from dysferlin-deficient patients [17] and from subjects submitted to a high palmitic acid diet [20], suggesting that skeletal muscle can actively participate in inflammasome upregulation. However, localization of NLRP3 has not yet been investigated within skeletal muscle. We unambiguously showed that NLRP3 was produced in vivo within myofibres under inflammatory conditions. After acute ip LPS injection, NLRP3 was detected as stained clusters in myocytes, suggesting activation and formation of the inflammasome complex, which may reach up to 3 μm in diameter in vitro [21]. During chronic muscle inflammation, like that seen in mdx mice, additional NLRP3 staining was detected to some extent in inflammatory infiltrates (data not shown).

Since ApN exerts anti-inflammatory effects on muscle via miR-711 and because miR-711 inhibits target genes that could lead to the inflammasome pathway [11], we explored whether NLRP3 could be regulated by ApN and its miRNA mediator in muscle. Both ApN and miR-711 mimic actually inhibited NLRP3 expression in C2C12 myocytes, while miR-711 blockade had opposite effects and reversed the anti-inflammatory action of ApN. Qualitatively similar results were obtained in vivo by using muscle electrotransfer of ApN cDNA or pre-miR-711. Taken together, our data indicate that miR-711 mediates the ApN-induced inhibition of NLRP3 expression and thus NLRP3 transcriptional priming. Although Nlrp3 is not a target gene of miR-711, miR-711 indirectly represses NLRP3 through two mechanisms: (1) by inhibiting NF-κb (see Fig. 1), which is involved in NLRP3 priming as shown in macrophages [22, 23], and (2) by inhibiting its target gene FADD ([11] and this paper), which is also known to promote priming and activation of NLRP3 ([12] and our own data).

We next examined whether ApN was also able to downregulate the inflammasome in a context of severe and chronic inflammation in muscle. To this end, we chose the mdx mouse, a model of DMD, where marked inflammation exacerbates disease progression [4, 5]. Circulating ApN was markedly diminished in mdx mice [9, 24]. Replenishment of ApN due to transgenesis strikingly reduced muscle inflammation, oxidative stress as well as muscle damage while increasing global force and endurance in mdx-ApN mice [9]. Herein, we further showed that ApN also reduced the marked (fourfold) rise of Nlrp3 mRNA and protein in mdx mice, thereby contributing to its anti-inflammatory action. This upregulation of Nlrp3 expression in DMD is at variance with another study conducted in a small number of animals [17]. When compared to NLRP3, miR-711 levels exhibited a reverse pattern of expression: a downregulation in mdx mice, in line with the decrease in circulating ApN, while an upregulation was observed in mdx-ApN animals. Thus, ApN downregulates NLRP3 expression likely via miR-711 in dystrophic mice. Moreover, as this hormone was also found to reduce mature IL-1β protein levels in muscle of these mice [9], this reinforces the concept mentioned above that ApN inhibits both NLRP3 priming and activation in muscle.

The role of NLRP3 in muscle needs to be unravelled. A very recent study has shown that muscle atrophy was attenuated in Nlrp3-KO mice made acutely septic by cecal ligation and puncture surgery. The polymicrobial sepsis was systemic [25]. To get a better insight into the role of NLRP3 in muscle inflammation, we investigated the impact of NLRP3 deletion in mdx mice, in which the inflammation originates from the muscle. We thus generated mdx mice with NLRP3 deletion. Each component of the NLRP3 inflammasome complex was overexpressed in regular mdx mice when compared to WT ones. Moreover, activation of the inflammasome was also enhanced in dystrophic muscle, as shown by higher caspase-1 activity and larger levels of mature IL-1β and IL-18. In mdx/Nlrp3-KO mice, caspase-1 activity and abundance of IL-1β/IL-18 were corrected. This contributed to normalize another major pro-inflammatory cytokine (TNFα) and an oxidative stress marker (PRDX3). Taken together, our data support an important role for NLRP3 in dystrophic mouse muscle. Yet, we used mice with a germline deletion of Nlrp3. We therefore cannot exclude the possibility that this systemic (i.e. non-muscle restricted) abrogation of NLRP3 with potential decreased circulating levels of IL-1β and IL-18 could participate in the attenuation of the dystrophic phenotype.

Eventually, we extended our work to humans. In basal conditions, NLRP3 was overexpressed in myotubes from DMD subjects compared to control ones, an effect which was attenuated by ApN treatment. MiR-711 levels exhibited a reverse pattern of expression. The upregulated NLRP3 mRNAs in DMD myotubes could result from the marked inflammatory context and the lower production of ApN by these dystrophic cells [19]. Reduced ApN production may in turn explain the lower expression of miR-711 [11]. Taken together, our data suggest that NLRP3 and ApN via miR-711 play an important role in pathogenesis of human DMD muscle.

Up to now, corticoid therapy is the only medication widely used in DMD [26]. Its beneficial effects have mostly been ascribed to reduced inflammation [27]. However, adverse effects must be considered: weight gain, growth retardation, Cushingoid appearance as well as bone demineralization, glucose intolerance and hypertension [28]. Delivery of miR mimics in total body muscles is still challenging. However, research on NLRP3 inflammasome inhibitors has developed tremendously over the past couple of years [15] with the recent discovery of two small specific inhibitory molecules (MCC950 [29] and the ketone body β-hydroxybutyrate [30]). NLRP3 inhibitors could therefore be alternate and/or additional candidates for DMD therapy, since NLRP3 is involved in the pathogenesis of this disease as well as in muscle inflammation. Moreover, targeting the inflammasome could also be beneficial to offset some of the side effects of glucocorticoids such as obesity and glucose intolerance, as NLRP3 overactivation plays a role in these metabolic disorders [16].

## Conclusions

NLRP3 is present in skeletal myofibres, where it is downregulated by ApN and its anti-inflammatory mediator, miR-711. The inflammasome NLRP3 is upregulated in DMD, and its ablation attenuates the dystrophic phenotype, suggesting that NLRP3 inhibitors may have a therapeutic potential for muscle inflammation and myopathies.

## Methods

### Animals

Several strains of mice were used. ApN-KO mice, which are characterized by a generalized lack of ApN, were obtained from Maeda et al. [31]. Transgenic mice overexpressing adiponectin have been generated in our lab: native full-length ApN was placed under the control of the adipocyte aP2 promoter, and because ApN is secreted, these mice showed a moderate elevation of circulating ApN levels [32]. Mdx mice (purchased from Jackson Laboratory, Bar Harbor, ME, USA) were crossed with these animals to obtain mdx mice overexpressing ApN (mdx-ApN mice) [9]. Mdx mice were also crossed with Nlrp3-KO mice (also from Jackson Laboratory) in order to obtain mdx Nlrp3-KO (mdx/Nlrp3-KO) mice (F2). In this study, we used four groups of mice (WT, Nlrp3-KO, mdx and mdx/Nlrp3-KO), which were all maintained on the same C57BL/6 J-10ScSn genetic background. In all experiments, only male mice were studied.

Mice were housed at a constant temperature (22 °C) with a fixed 12-h light, 12-h dark cycle. These animals received *ad libitum* a standard diet (Rat and Mouse No. 3 Breeding, Special Diets Services, Witham, UK) and were all studied at the age of 12 weeks, except for those from the experiment dealing with transgenic overexpression of ApN, which were studied at the age of 8 weeks [9].

At the end of the experiments, mice were sacrificed by cervical dislocation (between 09.00 and 11.00 h). Blood samples were saved. Pairs of *tibialis anterior* muscles were weighed, frozen in liquid nitrogen and stored at −80 °C for subsequent analyses.

All procedures were approved by the Ethical Committee for Animal Experimentation from the Medical Sector at the Catholic University of Louvain (No. LA1230396).

### Muscular electrotransfer of ApN gene or pre-miR-711 into muscle

#### *Expression plasmids and DNA preparation*

The plasmid encoding the ApN gene (p-ApN) was constructed by inserting the mouse full-length ApN cDNA [33] into the pcDNA™3.1D/V5-His-TOPO® vector (pcDNA™3.1 Directional TOPO® Expression Kit from Invitrogen, Thermo Fisher Scientific, Gent, Belgium) [11]. An empty plasmid was used as the control. The plasmid containing the precursor miRNA for *Mus musculus* miR-711 stem-loop with enhanced green fluorescent protein (EGFP) reporter gene (p-miR-711) as well as its negative control were purchased (GeneCopoeia, Rockville, MD, USA). All plasmids were amplified in *Escherichia coli* top 10 F′ (Invitrogen), purified with an EndoFree Plasmid Giga Kit (Qiagen, Venlo, The Netherlands) and then stocked at −80 °C.

#### *DNA injection and electroporation*

ApN-KO mice were submitted to muscle electrotransfer of the ApN gene or the pre-miR-711. Animals were anaesthetized with a mixture of ketamine (75 mg/kg body weight (BW); Ketalar, Pfizer, New York, NY, USA) and xylazine hydrochloride (10 mg/kg BW; Sigma-Aldrich, Bornem, Belgium) administered by ip injection. One *tibialis anterior* muscle was injected with 30 μl of a plasmid solution (1.5 μg/μl) coding for the ApN gene (p-ApN) or for the pre-miR-711 (p-miR-711), while the contralateral one received its respective control plasmid (p-ctrl). Muscles were then electroporated by using transcutaneous electric pulses (eight square-wave pulses of 200 V/cm and 20 ms per pulse at 2 Hz) that were applied by two 8-mm-spaced electrodes. Pulses were delivered by a Cliniporator system (Cliniporator, IGEA, Carpi, Italy), as described [11, 34–36]. Nine days after electrotransfer, ApN-KO mice were challenged or not by LPS (4 mg/kg BW; serotype 0127:B8 from *E. coli*; Sigma-Aldrich) and sacrificed 24 h later. Because LPS-injected mice reduced their daily food intake by 95% in our preliminary experiments, mice were fasted after the ip injection (LPS). In the muscle injected with the target plasmid, the expression of miR-711, which was measured by reverse transcription polymerase chain reaction (RT-PCR), was about four orders of magnitude higher than in the control muscle.

### In vivo studies of global force or resistance

Mice were submitted to three main tests as described previously [9].

#### Grip test

The grip strength test measures the muscle strength of combined fore-and hindlimb muscles. Limb strength was recorded using a grid connected to a sensor (Panlab-Bioseb, Vitrolles, France). The mice were gently laid on the top of the grid so that their four paws could grip the grid. The mice were then pulled back steadily until the grip was released down the complete length of the grid. Each test was repeated three times per day at an interval of 20 min for 3 days. Results are presented as the mean of the three values of force recorded on the last day and related to body weight [37].

#### Wire test

Animals were suspended by their forelimbs from a 1.5-mm-thick, 60-cm-long metallic wire at 45 cm above soft ground. The time (seconds) until the mouse completely released its grasp and fell down was recorded. Three trials were performed per day, with a 20-min recovery period between trials for a period of 3 days. The maximum time per trial was set to 180 s. Results are presented as the mean of the falling time of the three trials performed on the last day.

#### Eccentric exercise

The mice were subjected to a downhill running exercise on a treadmill with a downward inclination of 15° at a speed of 10 m/min for 10 min. This training was repeated daily for 3 days, and the mice were sacrificed 1 h after the 3rd session. Results are presented as the distance travelled by the mice on the last day.

### Immunohistochemistry and morphometry

Muscle samples were fixed in 10% formalin for 24 h and embedded in paraffin. Sections 5 μm thick were stained with hematoxylin. Sections were processed as previously described [9, 11, 36] using rabbit polyclonal antibodies directed against PRDX3 (dilution 1:350, incubation 2 h 30, a gift from B. Knoops, University of Louvain, Brussels, Belgium [38]); TNFα (1:100, 2 h 30, Cat# ab 6671, Lot# GR235155–1, RRID: AB_305641); IL-1β (1:300, 2 h 30, Cat# ab 2105, Lot# GR24376–3, RRID: AB_302842); IL-18 (1:100, 2 h 30, Cat# ab 71,495, Lot# GR310115–1, RRID:AB_1209302) — all from Abcam, Cambridge, UK. The antibody directed against IL-1β detected its bioactive form according to the manufacturer, while that directed against IL-18 recognized the total form. Rat monoclonal antibody directed against NLRP3 (1:100, 2 h 30, Cat# MAB 7578, Lot# CGSM0114021) was also used (from R&D Systems, Abingdon, UK). Before immunostaining, sections were submitted to heat-mediated antigen retrieval using a microwave oven and Tris-citrate buffer (pH 6.5). Binding of antibodies was detected by applying for 30 min at room temperature a second antibody, which was a biotinylated goat anti-rabbit IgG (H + L) or a biotinylated rabbit anti-rat IgG (H + L), both from Labconsult, Brussels, Belgium. Peroxidase activity was revealed with DAB (Thermo Fisher Scientific), which produces a brown staining. For each marker, all slides from each *tibialis anterior* were treated simultaneously for immunohistochemistry analysis and DAB revelation and then analysed together. Immunohistochemical controls were performed by omission of the first antibody or of the first and second antibodies or by using pre-immune serum. For quantification, whole muscle sections were scanned using the Leica SCN400 slide scanner (Leica Microsystems, Diegem, Belgium), and then the percentage of DAB surface area within muscle fibres was quantified using the Tissue Image Analysis 2.0 software (Leica).

### Western blotting

Skeletal muscle or cells were homogenized in a lysis buffer (Cell Signaling Technology, BIOKE, Leiden, The Netherlands) supplemented with 100 mM NaF and 1% protease inhibitor cocktail (Active Motif, Rixensart, Belgium). Immunoblotting was performed as reported [9] by using rabbit monoclonal antibodies directed against caspase-1 (Cat# LS-C138140–100, Lot# 65626, RRID:AB_10947747; from Bio-Connect, Huissen, The Netherlands) or the NLRP3 antibody used for immunochemistry. Signals were revealed by enhanced chemiluminescence, then quantified and normalized to actin levels using the ImageJ program (National Institutes of Health, Bethesda, MD, USA).

### Cell culture and transfection of miR mimic, anti-miR or siRNA in vitro

#### Culture of murine C2C12 cell line

C2C12 myoblasts were cultured as previously described [11]. Briefly, after proliferation, cells were cultured in basal medium (high glucose Dulbecco’s modified Eagle’s medium (DMEM) + 2% heat-inactivated horse serum (HS)) for 5 days to induce myogenic differentiation. Myotubes were used at day 5. Myotubes were pre-treated or not with 5 μg/ml mouse full-length recombinant ApN (Biovendor GmbH, Heidelberg, Germany) added to the basal medium for 24 h, while being challenged by 1 μg/ml of ultrapure LPS from *E. coli* K12 (Invivogen, Toulouse, France) for the last 20 h. At the end of the culture, cells were washed in ice-cold phosphate-buffered saline (PBS) before RNA or protein extraction.

#### Culture of human control and DMD myotubes

Primary cultures of human skeletal muscle cells were initiated from satellite cells obtained from DMD (*n* = 5) or healthy (*n* = 5) subjects via Myobank-AFM (Association Française contre les Myopathies).

Cultures were performed as described [19]. Myoblasts were grown in 35-mm plates at 37 °C in the presence of 5% CO_2_ in F-12 (Ham’s Nutrient Medium) supplemented with 20% foetal bovine serum (FBS), 1% l-glutamine and 100 μg/ml Primocin™ (Invivogen) (all other products from Thermo Fisher Scientific). After proliferation, at the end of which the seeding density had reached 70–80%, the growth medium was replaced by the fusion medium which consisted of 1 part DMEM, 1 part F-12 (Ham’s), 2% HS, 1% l-glutamine and Primocin™. This fusion medium was then changed every 2 days, and differentiation was allowed to continue for 11 days (the time required to obtain mature myotubes with characteristic elongated and multinucleated morphology) before the experimentation period. The differentiation of DMD myoblasts was similar to that of the controls [19]. At the end of the experiments, cells were collected and rinsed twice in PBS before RNA extraction. All experiments involving human cells were performed in accordance with approved guidelines and regulations of the bioethics department of the Direction Générale de la Recherche et de l’Innovation, France (DGRI; No. AC-2013-1868).

#### Transfection of miR-711 mimic, miR-711 inhibitor or FADD siRNA in C2C12 cells and/or human myotubes

Synthetic double-stranded oligonucleotide mimicking mature endogenous mouse or human miR-711 (miR-711 mimic, 5 nM), miR-mimic negative control (AllStars Negative Control, ctrl+, 5 nM), Anti-miR-711 mouse or human single-stranded oligonucleotide (Anti-miR, 50 nM) or Anti-miR negative control (miScript Inhibitor Negative Control, Ctrl–, 50 nM) (all from Qiagen) were delivered into respective species of mature myotubes. Delivery was performed by using Dharmafect 3 siRNA transfection reagent (Dharmacon, Lafayette, CO, USA). Cells and media were collected 24 h (mimic) or 28 h (anti-miR) post transfection. In some experiments, 4 h after initiation of transfection with Anti-miR or its negative control, cells were treated with or without mouse or human recombinant ApN (5 μg/ml) for 24 h (Biovendor), while being challenged or not by LPS (1 μg/ml; C2C12 cells) or by human recombinant TNFα + IFNγ (both at 10 ng/ml; human cells) for the last 20 h.

In some experiments, C2C12 cells were transfected with either the On-Targetplus Non-targeting pool siRNAs (negative control, NT siRNAs), or a specific On-Targetplus siRNA SMARTpool against FADD (50 nM) (all from Dharmacon) for 24 h by using Lipofectamine RNAiMAX Transfection Reagent (Thermo Fisher Scientific). Cells were then treated with LPS as described above for the last 20 h.

### Direct miRNA or mRNA quantification by RT-quantitative PCR (qPCR)

We isolated miRNA and RNA from cultured cells with TriPure reagent (Sigma-Aldrich).

For miRNA quantification, 1 μg total RNA was reverse transcribed by using the NCode™ VILO™ miRNA cDNA Synthesis Kit (Thermo Fisher Scientific). We amplified 10 ng of total RNA equivalents with iQSyber Green Supermix (Bio-Rad Laboratories, Temse, Belgium) using commercial miRNA-specific forward primers (Qiagen) and a reverse universal primer (provided in the NCode VILO miRNA cDNA Synthesis Kit).

For mRNA quantification, 2 μg of total RNA was reverse transcribed as described previously [11, 39], and qPCR was performed with designed primers (Additional file 3: Table S1).

The threshold cycles (Ct) were measured in separate tubes and in duplicate. To ensure the quality of the measurements, each plate included a negative control for each set of primers, and analysis of the melting curve was carried out at the end of the amplification. Cyclophilin (mouse) and TATA-box-binding protein (TBP, human) were used as reporter genes. Relative changes in the expression level of one specific gene were presented as 2^-ΔΔCt^ [11, 39].

### Presentation of the results and statistical analysis

The results are means ± standard error of the mean (SEM) for the indicated numbers of mice or independent cultures. Individual data for each experiment are provided as Additional file 4. Electroporated muscle groups were compared using a two-tailed paired Student’s *t* test. Comparisons of the three groups of mice (WT, mdx and mdx-ApN) were performed by one-way analysis of variance (ANOVA) followed by Tukey’s test (Prism 6; GraphPad Software, La Jolla, CA, USA). Comparisons of the four groups of mice (WT, Nlrp3-KO, mdx, mdx/Nlrp3-KO) were assessed by two-way ANOVA (influence of myopathy and NLRP3 depletion) with an *F* test, followed by *post hoc* two-by-two comparisons with a Bonferroni correction for multiple comparisons (Prism 6). The same statistical analysis was used to study the influence of ApN treatment and that of DMD genotype in human myotubes in vitro. Eventually, all other in vitro experiments were carried out using a two-tailed paired Student’s *t* test. Differences were considered statistically significant at *P* < 0.05.

## Additional files

Additional file 1: **Figure S1.** This figure shows a highly positive correlation between NLRP3 protein levels when measured either by Western blotting or immunostaining. (PDF 549 kb)

Additional file 2: **Figure S2.** FADD and TOLLIP are target genes of miR-711 in human DMD and C myotubes. (PDF 428 kb)

Additional file 3: **Table S1.** Primers used for RT-qPCR. (PDF 237 kb)

Additional file 4: Individual data values for all experiments are available in an Excel file. Data sets are sorted by figure, and an additional section is provided in order to recap *n* < 6 experiments. (XLSX 45 kb)

## Abbreviations

ApN: adiponectin
ApN-KO mice: adiponectin knockout mice
ASC: apoptosis-associated speck-like protein
BW: body weight
C: control
CK: Creatine Kinase
DAB: 3,3’-diaminobenzidine
DMD: Duchenne muscular dystrophy
FADD: Fas-associated protein with death domain
IFNγ: Interferon gamma
IL: Interleukin
ip: intraperitoneal
LPS: lipopolysaccharide
mdx mice: a murine model of DMD
mdx-ApN mice: mdx mice overexpressing ApN
mdx/Nlrp3-KO mice: mdx mice with Nlrp3 depletion
miRNA: micro RNA
NF-κB: nuclear factor kappa B
Nlrp3-KO mice: Nlpr3 knockout mice
NT: non-targeting
p-ApN: ApN cDNA containing plasmid
p-ctrl: control plasmid
p-miR-711: pre-miR-711 containing plasmid
PRDX3: peroxiredoxin 3
siRNAs: small interfering RNAs
TBP: TATA-box-binding protein
TNFα: tumor necrosis factor alpha
TOLLIP: Toll interacting protein
WT mice: Wild-Type mice
