# Supplementary material for: Downregulation of the NLRP3 inflammasome by adiponectin rescues Duchenne muscular dystrophy
Source: BMC Biol. 2018 Mar 20;16:33. doi: 10.1186/s12915-018-0501-z (PMC5861675; doi:10.1186/s12915-018-0501-z)

Fig. S1. Comparison of NLRP3 quantified by Western blotting or immunostaining. *Tibialis anterior* muscles were sampled from WT mice, Nlrp3-KO mice, mdx mice or mdx/Nlrp3-KO mice. NLRP3 protein was measured by using either Western blotting (like that shown on Fig.7) or immunostaining with subsequent measurement of DAB areas on muscle sections (like those shown on Fig.8). Data were expressed as percentages of WT values. As the signal was undetectable in KO mice, the correlation analysis using Pearson's test was performed only in non-KO mice (i.e. WT and mdx mice, n=12) from Fig. 7. There was a highly positive correlation between both techniques (r = 0.8982, P < 0.0001).

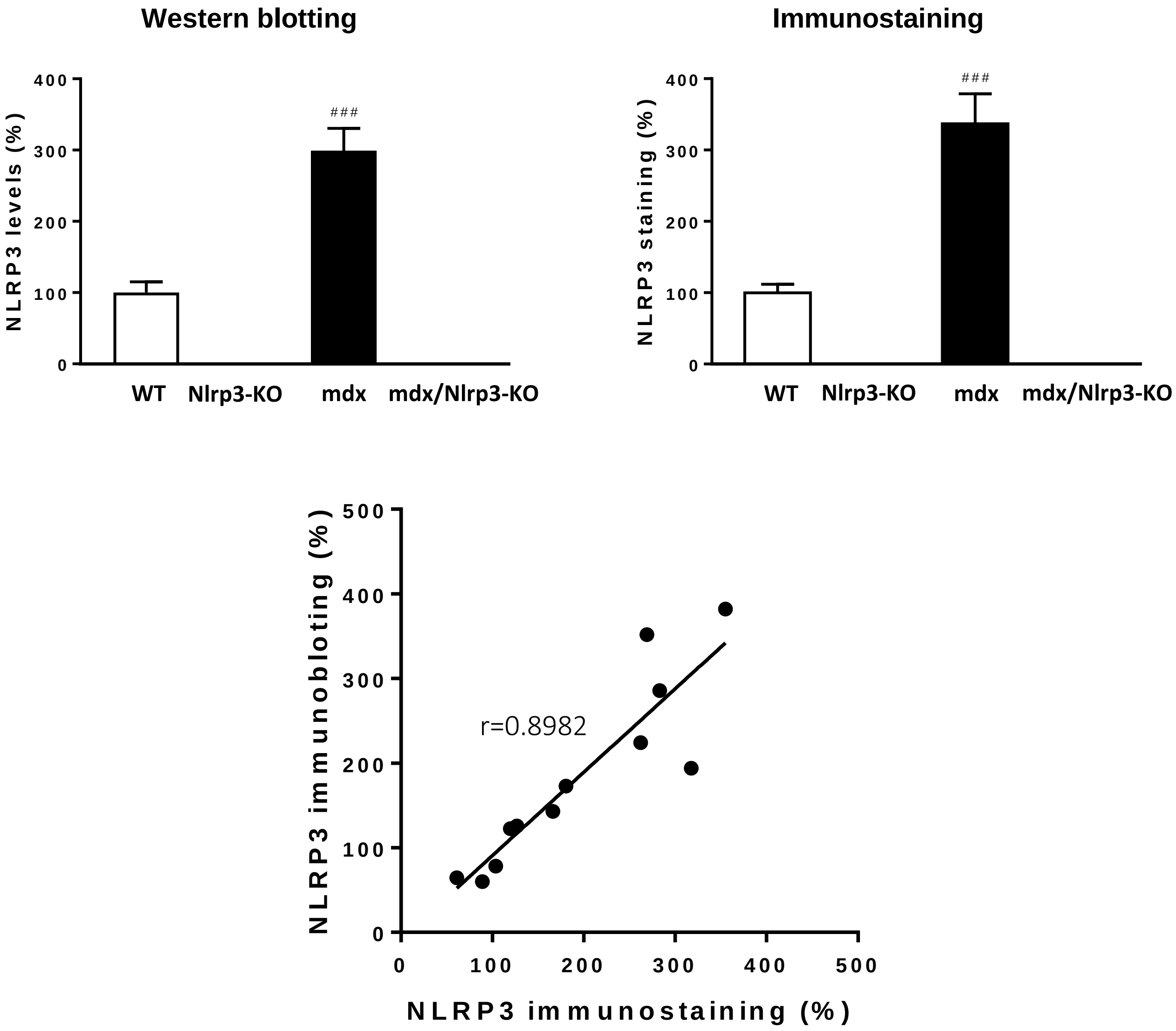

Supplement: Supplementary file 1 — Figure S1. This figure shows a highly positive correlation between NLRP3 protein levels when measured either by Western blotting or immunostaining. (PDF 549 kb) [file 12915_2018_501_MOESM1_ESM.pdf]
