# Supplementary material for: Downregulation of the NLRP3 inflammasome by adiponectin rescues Duchenne muscular dystrophy
Source: BMC Biol. 2018 Mar 20;16:33. doi: 10.1186/s12915-018-0501-z (PMC5861675; doi:10.1186/s12915-018-0501-z)

Fig. S2. Involvement of target genes of miR-711 in human DMD and C myotubes. Differentiated myotubes from C or DMD subjects were challenged by an inflammatory stimulus (a combination of TNFα and IFNγ), then transfected or not with miR-711 mimic or anti-miR-711 (or their respective controls: ctrl+ or ctrl-), while being or not treated by ApN (as described Fig. 9). mRNA levels of FADD and TOLLIP were normalized to TBP and presented as the relative expression compared to the basal condition (i.e. no inflammation, no transfection or any other treatments) represented by the dotted line. Values are means ± SEM for 7 independent cultures derived from 5 different subjects in each Control and DMD group. * $P < 0.05$, ** $P < 0.01$, *** $P < 0.001$ for indicated conditions.

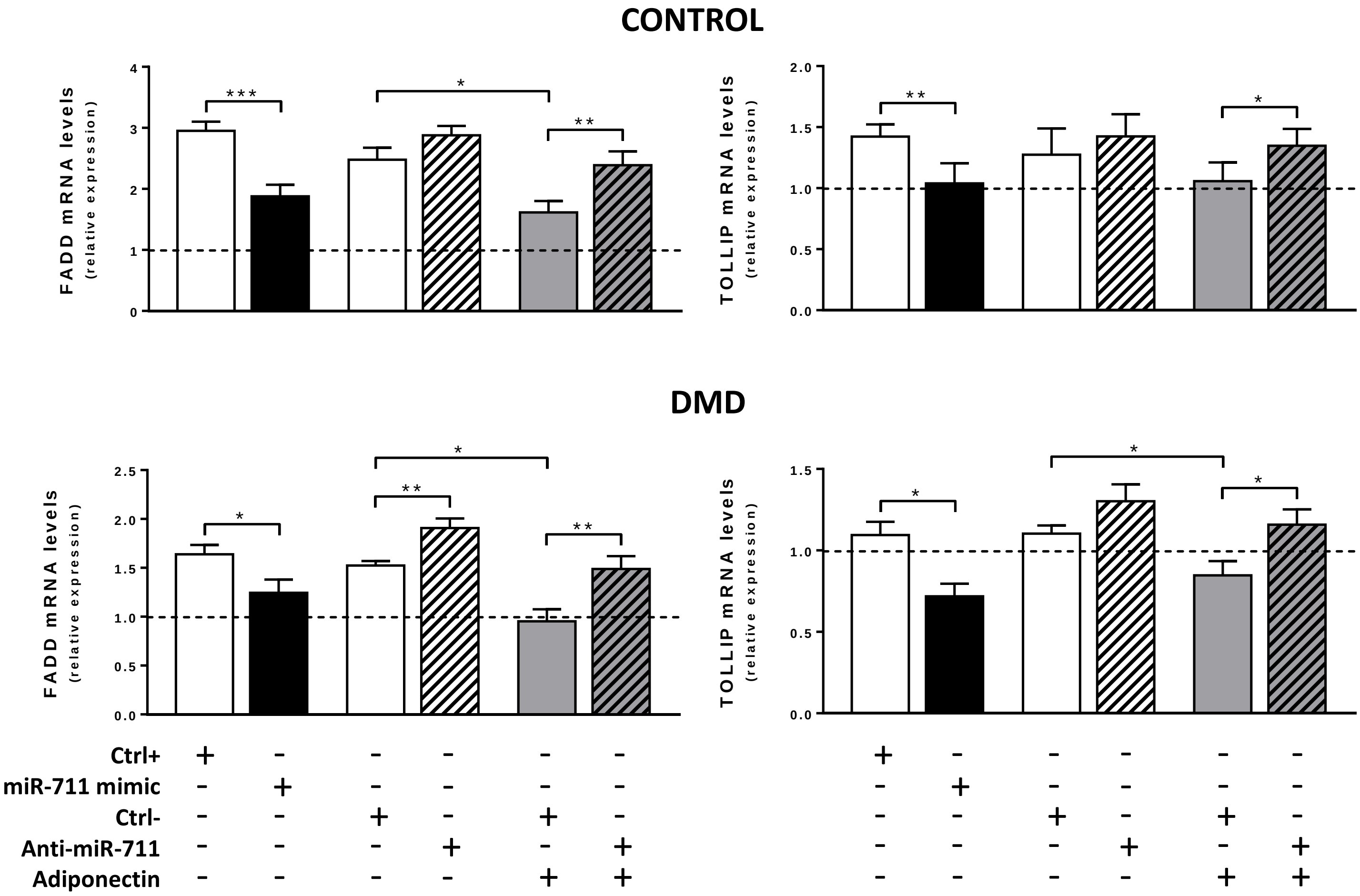

Supplement: Supplementary file 2 — Figure S2. FADD and TOLLIP are target genes of miR-711 in human DMD and C myotubes. (PDF 428 kb) [file 12915_2018_501_MOESM2_ESM.pdf]
