# Supplementary material for: Downregulation of the NLRP3 inflammasome by adiponectin rescues Duchenne muscular dystrophy
Source: BMC Biol. 2018 Mar 20;16:33. doi: 10.1186/s12915-018-0501-z (PMC5861675; doi:10.1186/s12915-018-0501-z)
Supplement: Supplementary file 3 — Table S1. Primers used for RT-qPCR. (PDF 237 kb) [file 12915_2018_501_MOESM3_ESM.pdf]

**Supplementary Table 1: Gene sequences used as forward and reverse primers for RT-qPCR**

| Gene | Forward Primer | Reverse Primer |
|---|---|---|
| **Mouse** | | |
| ASC | AGACATGGGCTTACAGGAGCTG | CCACAAAGTGTCCTGTTCTGGC |
| Caspase-1 | TACCTGGCAGGAATTCTGGAGC | CAGTCAGTCCTGGAAATGTGCC |
| IL-10 | GCATGGCCCAGAAATCAAGGAG | TCACTCTTCACCTGCTCCACTG |
| IL-18 | TGGCTGCCATGTCAGAAGACTC | CTGCGGTTGTACAGTGAAGTCG |
| IL-1β | GCTCTCCACCTCAATGGACAGA | GCCCAAGGCCACAGGTATTTTG |
| Nlrp3 | TCTGACCTCTGTGCTCAAAACCAAC | TGAGGTGAGGCTGCAGTTGTCTAAT |
| PRDX3 | GACATACTGTGGTCTGCCTCTG | CCTTTAAAATAGGGCGCGTG |
| TNFα | GCCACCACGCTCTTCTGTCT | GTCTGGGCCATGGAACTGAT |
| Cyclophilin | AACCCCACCGTGTTCTTC | TGCCTTCTTTCACCTTCCC |
| **Human** | | |
| FADD | CCTGGGGAAGAAGACCTGTGTG | TCGATGCTGTCGATCTTGGTGT |
| NLRP3 | GCTTCGACATCTCCTTGGTCCT | AGCTGACCAACCAGAGCTTCTT |
| TNFα | CTCTTCTGCCTGCTGCACTTT | GATGATCTGACTGCCTGGGC |
| TOLLIP | ATCACGGTGGTACAGGCAAAGT | TATTCCAGCGGGGATTCTTGGC |
| TBP | CCCCATGACTCCCATGACCC | ACGAAGTGCAATGGTCTTTAGGT |
